# Machine Learning–Based Overall Survival Prediction of Elderly Patients With Multiple Myeloma From Multicentre Real-Life Data

**DOI:** 10.3389/fonc.2022.922039

**Published:** 2022-06-30

**Authors:** Li Bao, Yu-tong Wang, Jun-ling Zhuang, Ai-jun Liu, Yu-jun Dong, Bin Chu, Xiao-huan Chen, Min-qiu Lu, Lei Shi, Shan Gao, Li-juan Fang, Qiu-qing Xiang, Yue-hua Ding

**Affiliations:** ^1^ Department of Hematology, Beijing Jishuitan Hospital, 4th Clinical Medical College of Peking University, Beijing, China; ^2^ Department of Hematology, Peking Union Medical College Hospital, Chinese Academy of Medical Sciences, Beijing, China; ^3^ Department of Hematology, Beijing Chao Yang Hospital, Capital Medical University, Beijing, China; ^4^ Department of Hematology, The First Hospital of Peking University, Beijing, China

**Keywords:** multiple myeloma, survival model, elderly patients, random survival forest (RSF), deep hit algorithms, cox proportinal hazards model (CPH), deep survival algorithms

## Abstract

**Objective:**

To use machine learning methods to explore overall survival (OS)-related prognostic factors in elderly multiple myeloma (MM) patients.

**Methods:**

Data were cleaned and imputed using simple imputation methods. Two data resampling methods were implemented to facilitate model building and cross validation. Four algorithms including the cox proportional hazards model (CPH); DeepSurv; DeepHit; and the random survival forest (RSF) were applied to incorporate 30 parameters, such as baseline data, genetic abnormalities and treatment options, to construct a prognostic model for OS prediction in 338 elderly MM patients (>65 years old) from four hospitals in Beijing. The C-index and the integrated Brier score (IBwere used to evaluate model performances.

**Results:**

The 30 variables incorporated in the models comprised MM baseline data, induction treatment data and maintenance therapy data. The variable importance test showed that the OS predictions were largely affected by the maintenance schema variable. Visualizing the survival curves by maintenance schema, we realized that the immunomodulator group had the best survival rate. C-indexes of 0.769, 0.780, 0.785, 0.798 and IBS score of 0.142, 0.112, 0.108, 0.099 were obtained from the CPH model, DeepSurv, DeepHit, and the RSF model respectively. The RSF model yield best scores from the fivefold cross-validation, and the results showed that different data resampling methods did affect our model results.

**Conclusion:**

We established an OS model for elderly MM patients without genomic data based on 30 characteristics and treatment data by machine learning.

## 1 Introduction

Multiple myeloma (MM) is an incurable neoplastic disease derived from abnormal plasma cells that predominantly affects elderly patients (1), and more than 60% of patients are over 65 years of age (2). Age has been deemed a prognostic factor and criterion of treatment regimen selection. MM patients ≥ 65 years are not candidates for autologous haematopoietic stem cell transplantation and show poor progression-free survival (PFS) and overall survival (OS) compared with patients younger than 65 years (3). The other limitation is that elderly patients usually do not meet the eligibility criteria of clinical studies, probably due to more comorbidities that cause higher Eastern Cooperative Oncology Group (ECOG) scores and lower estimated glomerular filtration rates (eGFRs). The application of novel agents, including bortezomib, lenalidomide and daratumumab, for nontransplant candidates recommended by the American National Comprehensive Cancer Network (NCCN) (4) is insufficient in the real world (5). Moreover, the elderly group was highly heterogeneous compared with the young group, with poor biological characteristics and more adverse reactions, resulting in a poor treatment response and no subsequent treatment after frontline therapy. Improving outcomes in the elderly MM population is dependent on selecting an appropriate therapy strategy according to elderly patients’ specific prognostic stratification. Although there are many stratification systems, few survival prognostic models have been generated for elderly patients, especially using real-world data.

The widely used prognostic indexes are the International Staging System (ISS), the more recent revised ISS (R-ISS), and the International Myeloma Working Group (IMWG) recommendations for risk stratification derived from clinical trials (6–8). The concordance indexes (c-indexes) of the above stratification systems validated by real-world data range from 57% to 65% (9), revealing substantial room for improvement. In recent reports, machine learning, including deep learning and random forests, has been implemented in cancer prognosis prediction (10). Maria Victoria et al. created a 50-variable random forest model including 4 biochemical variables (age, ISS stage, β2-microglobulin and frontline regimen) and 46 gene expression variables (c-index 78%) (11). This model was also based on clinical trials and is not suitable for patients without genetic features. In addition, the treatment response and maintenance therapy also affect the OS of MM patients. In this study, we enrolled 338 elderly MM patients (age ≥65 years) from 4 centres in Beijing, China, and used machine learning methods to incorporate 30 parameters, such as baseline data, genetic abnormalities and treatment options, to construct a prognostic model for survival prediction.

Survival analysis includes a set of methods that analyses the expected duration and factors affecting the expected duration until one event occurs. Most commonly used statistical methods assume that this potential relationship follows certain distributions. For example, the Cox proportional hazards model assumes that the logarithm of the sample hazard rate is linearly related to the covariate, but in fact, it is difficult to determine the actual underlying relationships.

On the other hand, random survival forest (RSF) avoids making restrictive assumptions and is able to provide an unbiased estimate of the error rate even when there is missing data (12). Recently, researchers in the health care field have started to use RSF tools to analyse patient data (13, 14). There are also survival analysis studies being done in the deep neural network field. Farragi et al. first proposed the use of feedforward neural networks to study the relationship between variables and risk factors, and many subsequent studies extended their idea (15, 16). The DeepHit model emerged from this idea and learns the joint distribution of survival time and events directly, avoiding restrictive assumptions and time invariance (17).

In this study, we enrolled 338 elderly MM patients (age ≥65 years) from 4 centres in Beijing, China, and used the cox proportional hazards model (CPH); DeepSurv; DeepHit; and the random survival forest (RSF) model to incorporate 30 parameters, such as baseline data, genetic abnormalities and treatment options, to construct a prognostic model for OS prediction.

## 2 Methods

### 2.1 Patient Selection and Variable Acquisition

All geriatric newly diagnosed multiple myeloma (NDMM) patients aged 65 years and older were reviewed at the Department of Hematology of four hospitals from January 2016 to September 2020. Patients who received no treatment or lost to follow-up were excluded. We selected 338 data which had >80% full annotation for 30 variables including baseline characteristics (sex, age, GA score, and ECOG score), myeloma-specific factors [haemoglobin, calcium, albumin, eGFR, M-spike, β2-microglobulin, LDH, ISS stage, RISS stage, and FISH detection including gain 1q21 and del 17p, t (11, 14), t (4, 14); t (14, 16), and t (14, 18)] and treatment condition (induction regimen, induction response, maintenance regimen, times for maintenance, and different treatment lines) (**Supplementary Table 1**). We defined 65 years old was the cut-off for elderly MM as patients ≥ 65 years old were not candidates for autologous hematopoietic stem cell transplantation and showed poor PFS and OS compared with patients younger than 65 (19). The OS was estimated from first treatment and censored at the last date at which they were known to be alive until September 30, 2020. The median follow-up was 27 months (1–60).

All of these study procedures were performed in accordance with the Declaration of Helsinki and were approved by the ethics committee of Beijing Jishuitan Hospital (201907–04). Written informed consent was obtained from each patient prior to data collection and analysis.

### 2.2 Data Preprocessing

#### 2.2.1 Data Cleaning and Standardization

The 338 data entries suffer from missing data problems. Different imputation methods were implemented for the variables based on their characteristics and our clinical knowledge. Detailed imputation methods for each variable were shown in **Supplementary Table 3**.

Simple imputation methods are widely used methods when dealing with missing data in health care studies (20). In this study, the observations were grouped according to the maintenance schema first, and continuous variables were imputed using the mean of its group. Group mean imputation can ensure that the mean of the variable in each group does not change after imputation. For variables with discrete values, we used hot-deck imputation and assumed that the data entries with similar survival time would have similar variable characteristics. Therefore, we sorted the data based on survival time, and discrete missing myeloma-specific factors were imputed using the corresponding value of the previous observation.

After data imputation, we addressed the problem that a large value range appeared between variables. To facilitate the training and convergence of the model, we first compressed the value space of each continuous variable to [0, 1] and then normalized it to form a dataset denoted as *D*.

#### 2.2.2 Data Resampling

Two data resampling methods were implemented before performing the tests.

The first method resamples the dataset *D* by a ratio of 7:1.5:1.5, giving us the dataset D^*^ = (*D_train_*, *D_valid_*, *D_test_*). *D_train_* contains 236 (70%) of the data points, while *D_valid_* and *D_test_* contain 51 (15%) each. Each data entry is a 31-dimensional vector. Based on this dataset, we conducted model building using four different methods. There was no difference between the three datasets by Mann–Whitney U nonparametric tests using SPSS 20.0 (SPSS, Inc., Chicago, IL) (**Supplementary Table 2**).

The result of the models might be affected by how the dataset was resampled because of our limited data size. Therefore, to further illustrate the effectiveness of the models and compare the pros and cons of the four models, we resampled the data with a second method and used fivefold cross-validation for model evaluation. The original data were divided into five equal subsets, and each subset was stratified and sampled while ensuring that the value range of the OS between the training set and the test set was roughly the same. After that, we took four of the subsets as the training set and the remaining one as the test set to train and test the four models. The dataset created here was denoted as *D* ^**^.

**Figure 1:**
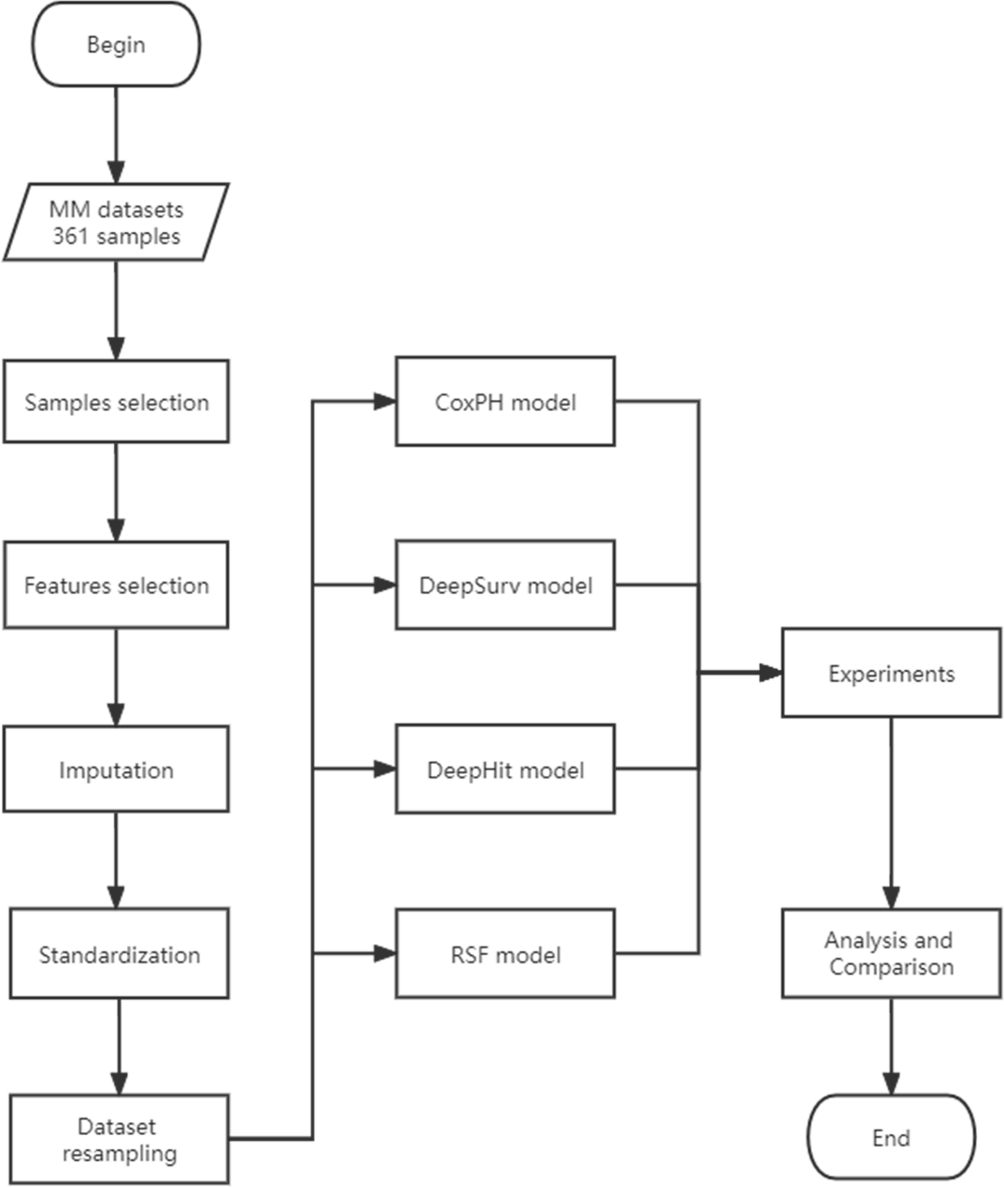
Data Analysis Flowchart.

A detailed data analysis flowchart is presented in **Figure 1**. R programming (R Core Team, Vienna, Austria) and Python v 3.6.7 (Python Software Foundation, Scotts Valley, USA) were used for the analysis in this paper.

### 2.3 Data Analysis

Four algorithms were selected to build models and analyse the influencing factors of the survival time: the cox proportional hazards model (CPH); DeepSurv; DeepHit; and the random survival forest (RSF).

#### 2.3.1 CPH Model

The Cox proportional hazards model is one of the most widely used models in survival analysis. It can be used to assess the influence of quantitative and categorical risk factors on survival time and make forecasts (19). In this study, we used the Python lifelines library to build a CPH model and forecasted survival times.

#### 2.3.2 DeepSurv Model

DeepSurv is a feed-forward neural network method based on the Cox proportional hazards model. The structure of DeepSurv is similar to the Faraggi-Simon network, and it can be used to model non-linear relationships between risk factors and survival time. DeepSurv has been proved to perform well on clinical data with missing datapoints and without prior assumptions on the risk function (20). We performed a grid search with the Pytorch framework to find the optimized hyper-parameter for the DeepSurv model in this study.

#### 2.3.3 DeepHit Model

The DeepHit model was originally designed for analysing the competing risk of multiple events (18). In this study, we only considered a single event, which was patient survival. Therefore, we can use a simplified DeepHit model to analyse our data. Through the softmax layer of the model, we can obtain an estimated probability sequence { *y*_1_,*y*_2_⋯,*y*_*T*_*max*__ }, where *y_t_* represents the probability estimate of the patient’s death at time t. While ensuring that $\sum_{i=1}^{T_{max}}y_{1}=1$ , the estimated survival rate of the patient at each time point can be obtained according to $\hat{P}\left(t=t^{*}\right)=1-\sum_{i=1}^{t^{*}}y_{i},t^{*}=1,2,3,\cdots ,T_{max}$ , and the survival curve can then be drawn.

Because the DeepHit model is designed to deal with discrete survival time, the event time is discretized using an isometric grid between the minimum duration and the maximum duration in the dataset. The isometric grid was set to one day in this study. The loss function of DeepHit contains two parts as shown in equation 1:


(1) $$L_{total}=\alpha *L_{1}+\left(1-a\right)*L_{2}$$

The hyper-parameter *α* is used to set the proportion of each loss. *L*_1_ is the negative log likelihood of the model, as shown in equation 2:


(2) $$L_{1}=\sum_{i}^{N}\left[I\left(k^{i}=1\right)*\text{log}\left(y_{t^{i}}^{i}\right)+I\left(k^{i}=0\right)*log\left({\hat{P}}_{k=1}\left(t^{i}|x^{i}\right)\right)\right]\;$$

where *I*(·) denotes the indicator function, and *N* denotes the sample size.

The idea of *L*_2_ came from the concordance index, and the calculation method is shown in equation 3:


(3) $$L_{2}=\;\sum_{i\neq j}A_{i,j}*\eta \left({\hat{P}}_{k=1}\left(t^{i}|x^{i}\right),{\hat{P}}_{k=1}\left(t^{i}|x^{j}\right)\right)$$

Where $A_{i,j}≜\;I\left(k^{i}=1,t^{i}<t^{j}\right),\eta \left(x,y\right)=exp\left(\frac{y-x}{\sigma }\right),\sigma$ denotes the hyper-parameter.

We performed grid search with the Pytorch framework to find the optimized hyper-parameter *α, σ*, and trained the DeepHit model.

#### 2.3.4 RSF Model

The RSF model is similar to the general random forest model, while the main difference is that the basic unit of RSF is a binary survival tree (18). Unlike traditional decision trees, survival trees usually use log-rank scores to maximize survival differences and use it as a criterion for splitting tree nodes. The final evaluation standard is the consistency index (18). Due to the limited data size and feature dimension, pruning and feature selection were not performed in this study.

The randomForestSRC package was used to build an RSF model for data training. The number of trees in the forest was set to 1000, the feature importance ranking was obtained, and the c-index indicator was used to evaluate the model performance.

### 2.4 Model Performance Evaluation

In order to compare the performance of the four models, we measured the Harrell concordance index (C-index) and the integrated Brier score (IBS).

#### 2.4.1 Concordance Index

The C-index is one of the most common indicators used in survival analysis. It is a generalization of the area under the ROC curve (AUC) (18), and represents the percentage of accurately-predicted patient pairs. The calculation method of C-index of patient *i* and patient *j* is shown in equation 4:


(4) $$C_{index}=P\left\{\hat{S}\left(t_{i}|x_{i}\right)<\hat{S}\left(t_{j}|x_{j}\right)|t_{i}<t_{j}\right\}$$

Where $\hat{S}\left(t_{i}|x_{i}\right)$ represents the predicted survival time of patient *i*. *C_index_* has a value between 0 and 1, *C_index_ =* 1 indicates that the model makes a perfect prediction.

#### 2.4.2 Integrated Brier Score

In multi-classification problems, the Brier score is defined as the average variance between predicted value and true value as shown in equation 5 (21):


(5) $$BS=\frac{1}{N}\sum_{i=1}^{N}\sum_{j=1}^{L}{\left({\hat{y}}_{ij}-y_{ij}\right)}^{2}$$

Where *N* denotes the sample size, *L* the number of classes, ${\hat{y}}_{ij}$ the model predicted value, and *y_ij_* the real value.

When dealing with survival analysis that has censoring problems, the Inverse Probablity of Censoring Weighted (IPCW) (18) needs to be considered in calculating the Brier score. The calculation method is shown in equation 6:


(6) $$BS\left(t\right)=\frac{1}{N}\sum_{i=1}^{N}\frac{{\left(0-\hat{S}\left(t|x\right)\right)}^{2}}{\hat{G}\left(t_{i}|x\right)}I\left(t_{i}\leq t,\delta_{i}=1\right)+\frac{{\left(1-\hat{S}\left(t|x\right)\right)}^{2}}{\hat{G}\left(t|x\right)}I\left(t_{i}>t\right)$$

Where $\hat{G}\left(t|x\right)$ is the Kaplan-Meier estimator, *δ* is the censoring indicator, and $\hat{S}\left(t|x\right)$ is the estimate of the survival function. Then we integrate the Brier score to get the integrated Brier score (IBS) as shown in equation 7:


(7) $$IBS=\int_{\text{min}\left(t\right)}^{\max\left(t\right)}BS\left(t\right)dt\;$$

## 3 Results

### 3.1 Clinical Characteristics of the Cohort

A summary of the baseline characteristics and treatment conditions of the patients in the cohort is presented in **Supplementary Table**. The median age was 70 years (65–86). Proteasome inhibitors (PIs), including bortezomib and ixazomib, were the most common induction regimen (64.5%), and PIs in combination with immunomodulatory drugs (IMiDs) were the second most common first line of therapy (18.4%). Few patients received IMiD-based (14.2%) and traditional regimens (2.9%). Of note, 20.4% of patients had a lower eGFR (<30 ml/min per 1.73 m), and 57.7% had an ECOG score higher than 2 at baseline.

### 3.2 Model Analysis

As mentioned above, we used the training set and validation set of *D* ^*^ to find the value of hyper-parameters in DeepSurv and DeepHit models. In order to minimize the influence on model performance by data resampling, we further tested model performances using the five-fold cross-validation with *D* ^**^.

#### 3.2.1 Model Parameter Tuning and Visualization

The best parameters we obtained from the training and the validation set of *D* ^*^ are: DeepSurv: Layers=3, Nodes per layer=32, dropout=0.4, learning rate = 0.003; and DeepHit: Layers=3, Nodes per layer=[32,32,60], dropout=0.4, learning rate = 0.0002, *α* = 0.1, *σ* = 0.3.

Based on these parameters, the training set *D_train_* from *D* ^*^ was used to build four models. CPH and DeepSurv models did not yield results that are as good as RSF or DeepHit, so we further analysed the RSF and the DeepHit models.

**Figure 2:**
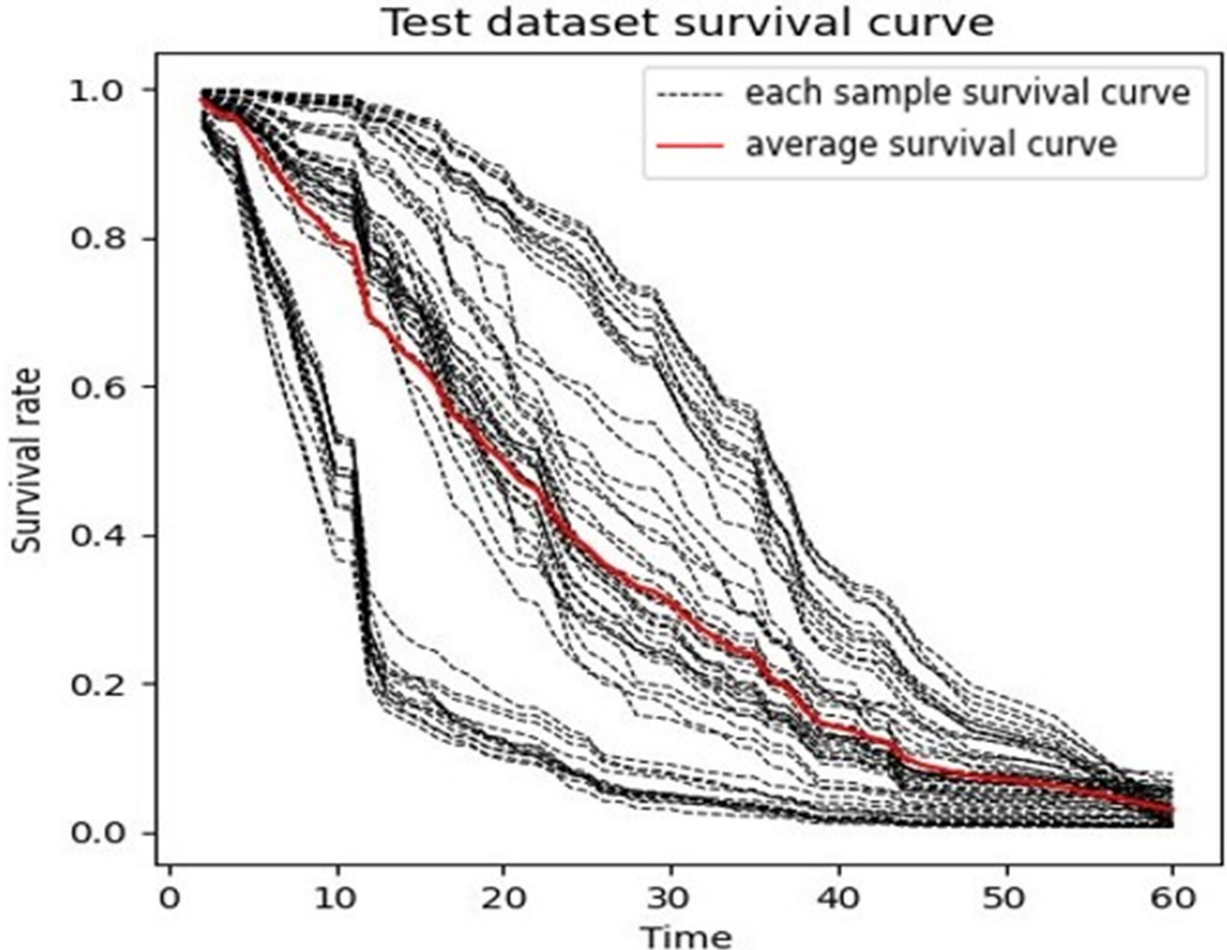
Predicted Survival Curve (Random Survival Forest).

**Figure 2** shows one RSF model that had a result near to average. The graph presents the predicted survival curve of the patients in the test set, where each dotted line represents the predicted survival curve for one patient, and the thick red line represents the average survival curve.

**Figure 3:**
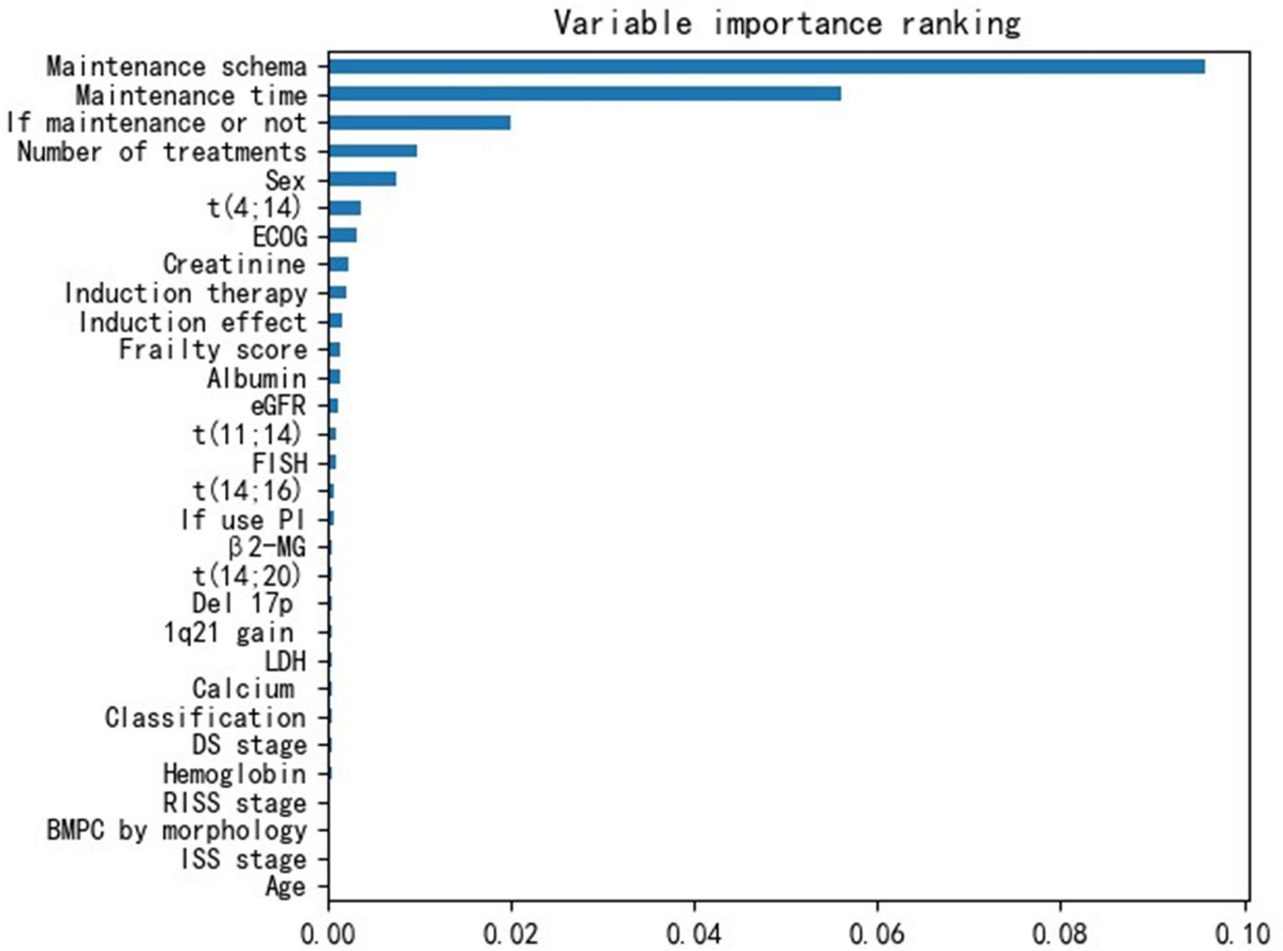
Variable Importance Ranking.

Additionally, we obtained the variable importance ranking based on their influences on the OS rate. **Figure 3** shows that the three variables related to the maintenance schema had the strongest influences on survival. This indicates that in the actual treatment of MM, maintenance therapy is very important.

**Figure 4:**
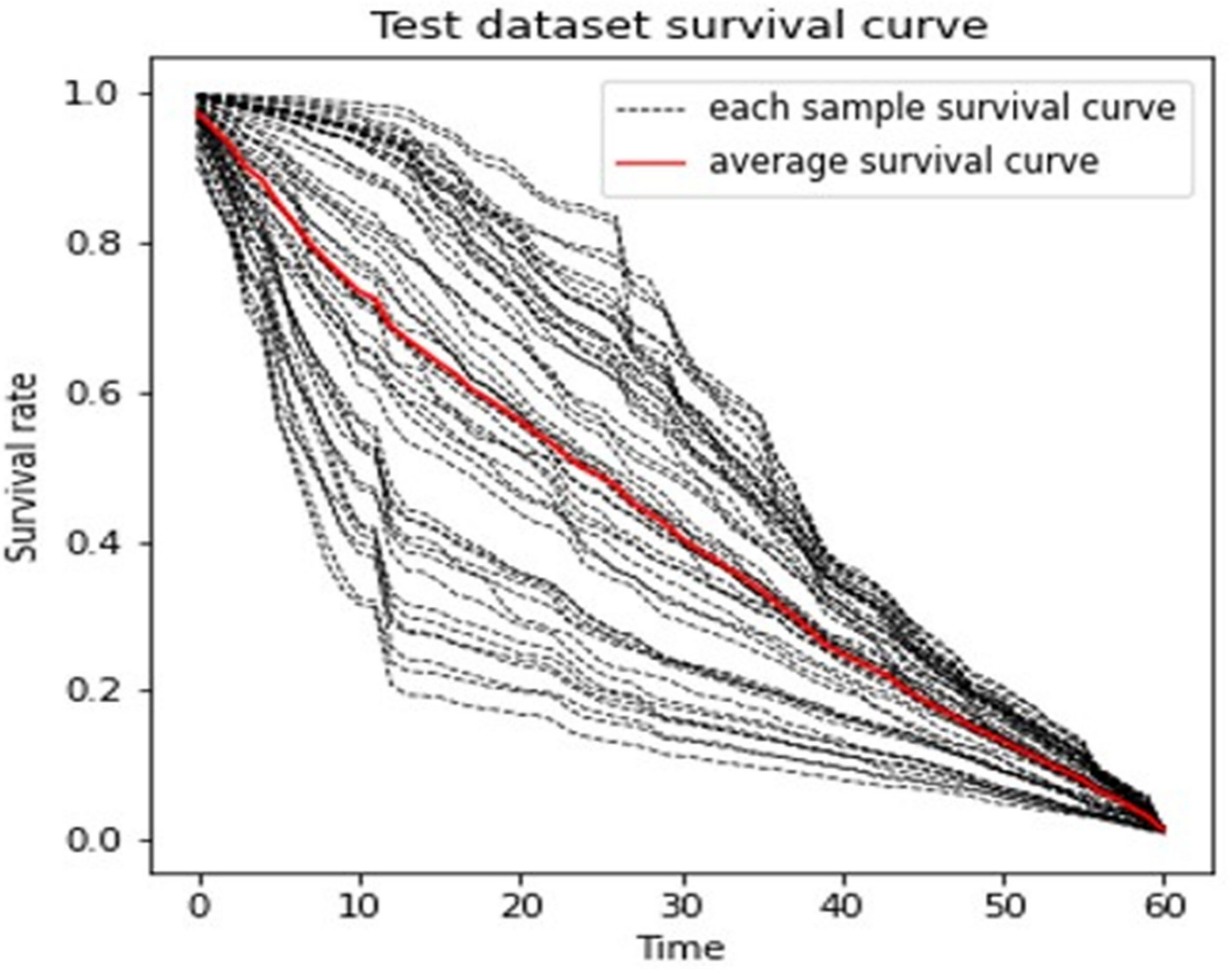
Predicted Survival Curve (DeepHit).

The DeepHit neural network model of a single event was built to discover information from more feature variables. **Figure 4** shows one model that had a result near the average under this case. The predicted survival curves of the patients in the test set are displayed.

**Figure 5:**
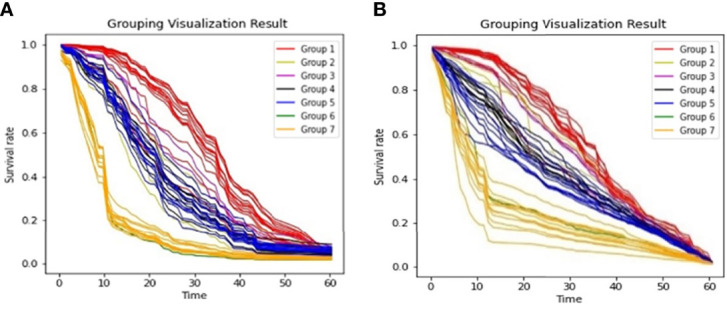
Survival Curves by Maintenance Schema **(A)**. Grouping Visualization Result of RSF; **(B)**. Grouping Visualization Result of DeepHit.

From **Figure 3**, we observed that the results are largely affected by the maintenance schema variables. Therefore, we generated a groupwise survival curve graph based on these variables. The results are shown in **Figure 5**, with the left subgraph presenting the RSF model and the right subgraph presenting the DeepHit model. It is obvious that the classification results of both models are acceptable. For the data entries with maintenance schema groups 1, 5, and 7, which represent maintenance treatment with IMiDs, no maintenance treatment due to relapse and refractory disease, and still in induction treatment, respectively, the model can effectively distinguish them; however, the classification effect of groups 2, 3, 4, 5, and 6 is not strong. The reason might be that the sample size of these indistinguishable groups is too small; for example, there is only one sample in group 6 in the test set. Another reason might be that when the missing values of the data were filled, a strong correlation between samples was introduced. Hence, it is difficult for the model to distinguish some of the groups.

We then calculated the variance of each group to characterize the degree of aggregation of the survival curves. The formula we used to calculate the variance of the curve is shown in equation (8), where *T_max_* denotes the maximum survival time, *count_k_* denotes the number of curves in the *k^th^* group, and $P_{i}^{k}$ denotes a probability sequence $\left\{P_{i,2}^{k},P_{i,2}^{k},\cdots ,P_{i,count_{k}}^{k}\right\}$ ; *std*(·) represents the variance function.


(8) $$\sigma_{k}=\;\;\left(\sum_{i=1}^{T_{max}}std\left(P_{i}^{k}\right)\right)/\;T_{max}$$

The calculation results are shown in **Table 1**. All the variances are less than 0.09, indicating that the aggregation of both models is acceptable. Because of the limited data size and the unequal data entry distribution in each group, it is difficult for us to compare the aggregation effect between the DeepHit model and the RSF model. However, the results showed that the two models have convincing performance on OS curve prediction for different groups of patients.

**Table 1:** Variance of each Maintenance Schema group.

Groups	Maintenance Schema	*count_i_*	RSF	DeepHit
**Group 1**	Immunomodulator	**14**	0.0667	**0.0369**
**Group 2**	Proteasome Inhibitor	**3**	0.0859	**0.0515**
**Group 3**	PI+iMiDs	**2**	0.0249	**0.0037**
**Group 4**	No	**8**	0.0333	**0.0190**
**Group 5**	Disease Progression	**11**	0.0434	**0.0408**
**Group 6**	Death	**1**	0.0000	0.0000
**Group 7**	Inducing	**12**	**0.0217**	0.0417

The bold value was the best results compared among groups.

#### 3.2.2 Cross-Validation

The actual data resampling result might affect the performance of the models due to our limited data size. Therefore, to further evaluate the performance of the four models, fivefold cross-validation was performed for each model. That is, four subsets of the dataset *D* ^**^ were used as the training set, and the remaining subset was used as the test set. Five tests were carried out for each model. The average of the five test results was used as the final result of a model. This method can alleviate the impact of data resampling on model results and better demonstrate the model performance. **Table 2** shows the C-index results, averaged over the fivefold cross-validation folds.

**Table 2:** C-index for Cross-validation.

	Fold 1	Fold 2	Fold 3	Fold 4	Fold 5	Mean	SD
**CPH**	0.760	0.802	0.776	0.772	0.734	0.769	0.022
**DeepSurv**	0.760	0.821	0.754	0.773	0.792	0.780	0.024
**DeepHit**	0.785	0.810	0.770	0.767	0.795	0.785	**0.016**
**RSF**	0.816	0.811	0.753	0.784	0.824	**0.798**	0.026

The bold value was the best results compared among groups.

CPH and DeepSurv models did not perform well under this data resampling method as well. The reason that RSF and DeepHit models performed better might be that they have less requirements on the dataset, so that they can deal better with more complex survival data. Another reason that DeepHit performed better than DeepSurv might be that the loss function of DeepHit took concordance index into consideration.

In general, the results show that different data resampling methods have noticeable effects on the model results. One possible reason is the size of our dataset is limited, and there are missing values in our dataset. We can also conclude that RSF models are more susceptible to data quality because it is shown in the results that the standard deviation of the RSF model is larger than that of the DeepHit model. Cross-validation presented a C-index result different from our result in the model training section. The average RSF C-index result (0.798) was slightly better than the average DeepHit result (0.785), because RSF is more suitable for small sample size data analysis. At the same time, DeepHit yielded a smaller standard deviation of C-index (SD = 0.016) comparing to RSF (SD = 0.026), because part of its loss function was designed based on the concordance index.

Moreover, **Table 3** presents the IBS for the four models under cross-validation. RSF presented a mean IBS of 0.099 and a standard deviation of 0.002, while DeepHit presented a mean of 0.108 and a standard deviation of 0.002. RSF has a better IBS (a value closer to 0) with a lower standard deviation, so the accuracy and stability of the RSF model are better based on IBS.

**Table 3:** IBS for Cross-validation.

	Fold 1	Fold 2	Fold 3	Fold 4	Fold 5	Mean	SD
**CPH**	0.1409	0.1341	0.1160	0.1644	0.1522	0.1415	0.0164
**DeepSurv**	0.1154	0.1070	0.1032	0.1174	0.1147	0.1115	0.0055
**DeepHit**	0.1092	0.1085	0.1086	0.1100	0.1041	0.1081	0.0021
**RSF**	0.0974	0.0964	0.0991	0.1001	0.1009	**0.0988**	**0.0017**

The bold value was the best results compared among groups.

Overall, the RSF model presented better discriminatory accuracy and provided the best model results on the elderly MM patient dataset.

## 4 Discussion

Due to the strong heterogeneity of MM, although there are many traditional assessment methods, such as ISS, RISS, chromosomal abnormalities and CIC, they still cannot meet clinical needs. There are also previous studies on machine learning methods in the MM field. Terebelo et al. reported a tool based on 3011 patients with NDMM from multiple centres in the US by multivariable Cox regression using weighted observations, achieving c-indexes of 64.7%-69.8% (21). In another recent report, Maria Victoria et al. developed a random forest model including the characteristic and GEP data of 730 patients for OS prediction with good discrimination (c-indexes of 0.818 and 0.780 in training and validation sets) (11). Groups from India proposed k-adaptive partitioning derived simple stage system using five baseline parameters and validated higher values of C-index on both MMIn and MMRF datasets, which outperformed ISS for OS calculation but was equivalent in the prognosis of PFS (22). However, most of these data came from clinical trials, and their role in real-world MM predictions, especially in older patients, is unclear (23).Although there have also been researches that applied machine learning and deep learning algorithms to build models and make survival predictions using real-world oral cancer (24, 25), breast cancer (26) and glioblastoma (27) patient data, the implementation of these methods on elderly MM patient data have not been fully discussed.

In this study, we presented feasible machine learning models for predicting the OS of elderly MM patients based on baseline clinical, biochemical, and treatment data. Our deep learning and random forest model involved 30 parameters, which combined frontline and maintenance treatment information, and achieved a high c-index of 80%. The RSF model presented the best model results on our dataset. Although the number of people in this study is small, all of them are elderly and represent multicentre data in the real world. Therefore, this model may provide dynamic prediction during the whole process of MM.

The visualization results showed that the use and prolonged use of maintenance therapy are critical for OS in MM, and the most commonly used maintenance therapy is lenalidomide. Real-life data from the US showed that approximately 50% of nontransplant patients do not receive follow-up therapy after first-line therapy (28). Similarly, many elderly Chinese patients do not receive maintenance therapy for various reasons, such as poor compliance, multiple comorbidities, poor physical fitness, and economic conditions.

Both ECOG and frailty scores had higher contribution rates, indicating the importance of applying performance status scores in elderly patients. The high attrition rate also suggests that choosing the optimal frontline treatment is crucial for prolonging OS in elderly MM patients. The recommended treatment regimens for MM patients ineligible for autologous stem cell transplantation (ASCT) include VRD, DaraRD, Rd and PCD (4). Over 80% of elderly MM patients in our group received PI and PI+IMiD-based first-line regimens in accordance with their treatment status in first-tier cities of China. Although DaraRD has been reported to improve PFS in patients ineligible for ASCT compared with RD (34.4 m vs. not reached) (29), the application of Dara was not extensive. Compared with survival model reported by previous studies (11, 21), the model derived from our real-life data is suitable for elderly MM patients without genomics data who received first-line therapy without daratumumab, so it will be easy applicable in real world.

One limitation of our study arises at the data imputation step. Although the current imputation methods we use are commonly used in health care studies, we are aware that more advanced imputation methods exist and might be able to lead to better results. We are planning to further discuss the influence of imputation methods on model results in our future studies.

What is more, due to the limited sample size, the results from our models are not stable enough. We will continue to enlarge the data size and improve the data quality. We believe that our model has a very good function and can reveal the relationship between different variables more clearly. We aim to provide credible and accurate reference guidance for medical clinical treatment with our models.

In conclusion, this work utilized all process variables, including baseline characteristics and treatment parameters, to provide a reliable OS prediction model for elderly MM patients. It is also suitable for patients without genomic testing and monoclonal antibody therapy due to economic and/or geographic constraints. The model is applicable to any disease stage, can be optimized on larger datasets, and can be used to select the appropriate intensity of treatment.

## Data Availability Statement

The raw data supporting the conclusions of this article will be made available by the authors, without undue reservation.

## Author Contributions

LB designed the study. Y-TW and LB analysed the data and wrote the manuscript, and all authors contributed to the interpretation of the data, prepared the manuscript, and approved the final version.

## Funding

This work was supported by Beijing JST Research Funding (grant no. XKXX-202111).

## Conflict of Interest

The authors declare that the research was conducted in the absence of any commercial or financial relationships that could be construed as a potential conflict of interest.

## Publisher’s Note

All claims expressed in this article are solely those of the authors and do not necessarily represent those of their affiliated organizations, or those of the publisher, the editors and the reviewers. Any product that may be evaluated in this article, or claim that may be made by its manufacturer, is not guaranteed or endorsed by the publisher.
