# Supplementary material for: Machine Learning–Based Overall Survival Prediction of Elderly Patients With Multiple Myeloma From Multicentre Real-Life Data
Source: Front Oncol. 2022 Jun 30;12:922039. doi: 10.3389/fonc.2022.922039 (PMC9293757; doi:10.3389/fonc.2022.922039)
Supplement: Supplementary file 1 [file Table_1.docx]

Sup Table 1. Clinical characteristics of patients in cohorts.

| Character | All patients (n=338) |
| --- | --- |
| Median follow-up |  |
| Age (y) | 70 (65-86) |
| Male/Female | 180(53.3%)/158(46.7%) |
| M protein |  |
| IgG | 146(43.2%) |
| IgA | 93(27.5%) |
| IgD | 15(4.4%) |
| light chain | 77(22.8%) |
| non-secreting/NA | 2(0.6%)/20(5.9%) |
| Calcium (mmol/l) | 2.32 (1.11-4.73) |
| CCr, umol/L | 87 (31-1370) |
| eGFR (ml/min per 1.73m) | 70.5(2-114) |
| <30 | 69 (20.4%) |
| Hemoglobin (g/L) | 96 (42-158) |
| LDH (IU/L) | 173(57~2048) |
| β2-MG (mg/L) | 5.4 (0.79-58.06) |
| ISS stage I/II/III/NA | 40/82/197/19 |
| R-ISS stage I/II/III/NA | 24/142/118/54 |
| BMPC by morphology (%) | 24.3 (0-98) |
| FISH | 275 (81.4%) |
| 1q21 gain | 126(45.8%%) |
| Del 17p | 43(15.6%) |
| t(4;14) | 28(10.2%) |
| t(11;14) | 43(15.6%) |
| t(14;16) | 6(2.2%) |
| t(14;20) | 1(0.4%) |
| ECOG ＞2 | 195 (57.7%) |
| GA score 2/1/0 | 141/57/140 |
| First line |  |
| IMiDs | 48 (14.2%) |
| PI | 218(64.5%) |
| PI+IMiDs | 62 (18.4%) |
| Other regimens | 10 (2.9%) |
| First line maitenance | 136 (40.2%) |
| IMiDs | 93 (27.5%) |
| PI | 26 (7.7%) |
| PI+IMiDs | 17 (5.1%) |
| Median maintenance (m) | 12 (1-50) |
