# Supplementary material for: Machine Learning–Based Overall Survival Prediction of Elderly Patients With Multiple Myeloma From Multicentre Real-Life Data
Source: Front Oncol. 2022 Jun 30;12:922039. doi: 10.3389/fonc.2022.922039 (PMC9293757; doi:10.3389/fonc.2022.922039)
Supplement: Supplementary file 2 [file Table_2.docx]

Sup Table 2. Variable Instances, Missing Instances, and Imputation Strategies

| Variable | Number of instances | Missing instances | Imputation strategy |
| --- | --- | --- | --- |
| Age (y) | 338 | 0 |  |
| Sex | 338 | 0 |  |
| Frailty score | 229 | 109 | Hot-Deck |
| ECOG | 338 | 0 |  |
| eGFR | 338 | 0 |  |
| Number of treatments | 338 | 0 |  |
| Maintenance schema | 338 | 0 |  |
| If maintenance or not | 259 | 79 | Hot-Deck |
| Maintenance time | 259 | 79 | Group Mean |
| Induction therapy | 338 | 0 |  |
| If use PI | 338 | 0 |  |
| Induction effect | 269 | 69 | Hot-Deck |
| Classification | 312 | 26 | Hot-Deck |
| DS stage | 338 | 0 |  |
| BMPC by morphology | 326 | 12 | Group Mean |
| Hemoglobin | 334 | 4 | Group Mean |
| Albumin | 206 | 132 | Group Mean |
| Calcium | 332 | 6 | Group Mean |
| Creatinine | 335 | 3 | Group Mean |
| LDH | 328 | 8 | Group Mean |
| β2-MG | 206 | 132 | Group Mean |
| ISS stage | 318 | 20 | Hot-Deck |
| RISS stage | 294 | 44 | Hot-Deck |
| FISH | 273 | 65 | Hot-Deck |
| 1q21 gain | 274 | 64 | Hot-Deck |
| Del 17p | 273 | 65 | Hot-Deck |
| t(14;16) | 265 | 73 | Hot-Deck |
| t(11;14) | 265 | 73 | Hot-Deck |
| t(4;14) | 265 | 73 | Hot-Deck |
| t(14:20) | 50 | 288 | Delete |
| event | 338 | 0 |  |
| Survival time | 338 | 0 |  |
